# Analysis of the mortality rate in patients admitted to the ICU for severe community-acquired pneumonia

**DOI:** 10.1186/cc14099

**Published:** 2015-03-16

**Authors:** C Joya-Montosa, MD Delgado-Amaya, H Molina-Diaz, E Curiel Balsera

**Affiliations:** 1 Hospital Regional de Málaga, Spain

## Introduction

The aim of the study was to analyze the factors associated with hospital mortality in patients with severe community-acquired pneumonia (CAP) who required ICU admission.

## Methods

An observational, retrospective study of patients with severe CAP admitted to the ICU between January 2008 and September 2013. We analyzed clinical, epidemiological and outcome variables. Quantitative variables were expressed as the mean and standard deviation. Qualitative variables are expressed as the percentage and absolute value. We applied the Mann-Whitney and Fisher's exact test, as needed, with an alpha error of 5%.

## Results

We analyzed 111 patients, 57.5 ± 17.7 years old, with 63.1% (70) males and APACHE II score on admission of 19.8 ± 17.7. ICU mortality was 29.7% (33) and in-hospital mortality was 32.4% (36). Ten percent of patients met criteria for medical care-associated pneumonia (HCAP); there were no significant differences in mortality between HCAP and CAP (*P *= 0.075). Patients chronically taking immunosuppressive therapy had a significantly higher mortality compared with the rest of the patients (47.8% vs. 28.4%, *P *= 0.07). The mortality rate was also higher in patients in whom NIV fail in the first 24 hours (42.9% vs. 17.6% with *P *= 0.09). Patients who required intubation and mechanical ventilation in the first 24 hours had a higher mortality rate (47.2% vs. 19%, *P *= 0.002). Regarding the etiology of pneumonia, in 11 patients the viral origin of infection was confirmed (10 patients had H1N1 pneumonia and one patient CMV pneumonia), with a mortality rate significantly lower than in patients with bacterial pneumonia (3.6% vs. 35.3%, *P *= 0.06). The use of the right antibiotic therapy at admission was associated with mortality (*P *= 0.0001).

## Conclusion

Patients admitted to the ICU with severe CAP and immunosuppressive therapy have higher mortality, with no differences between HCAP and CAP. The delay in intubation as well as bacterial and inappropriate antibiotic treatment are factors that increase mortality.

